# Antigen-presenting particle technology using inactivated surface-engineered viruses: induction of immune responses against infectious agents

**DOI:** 10.1186/1742-4690-4-32

**Published:** 2007-05-15

**Authors:** Joseph D Mosca, Yung-Nien Chang, Gregory Williams

**Affiliations:** 1 JDM Technologies, Inc., Ellicott City, MD 21042, USA

## Abstract

**Background:**

Developments in cell-based and gene-based therapies are emerging as highly promising areas to complement pharmaceuticals, but present day approaches are too cumbersome and thereby limit their clinical usefulness. These shortcomings result in procedures that are too complex and too costly for large-scale applications. To overcome these shortcomings, we described a protein delivery system that incorporates over-expressed proteins into viral particles that are non-infectious and stable at room temperature. The system relies on the biological process of viral egress to incorporate cellular surface proteins while exiting their host cells during lytic and non-lytic infections.

**Results:**

We report here the use of non-infectious surface-engineered virion particles to modulate immunity against three infectious disease agents – human immunodeficiency virus type 1 (HIV-1), herpes simplex virus (HSV), and Influenza. Surface-engineering of particles are accomplished by genetic modification of the host cell surface that produces the egress budding viral particle. Human peripheral blood lymphocytes from healthy donors exposed to CD80/B7.1, CD86/B7.2, and/or antiCD3 single-chain antibody surface-engineered non-infectious HIV-1 and HSV-2 particles stimulate T cell proliferation, whereas particles released from non-modified host cells have no T cell stimulatory activity. In addition to T cell proliferation, HIV-based particles specifically suppress HIV-1 replication (both monocytotropic and lymphocytotropic strains) 55 to 96% and HSV-based particles specifically induce cross-reactive HSV-1/HSV-2 anti-herpes virus antibody production. Similar surface engineering of influenza-based particles did not modify the intrinsic ability of influenza particles to stimulate T cell proliferation, but did bestow on the engineered particles the ability to induce cross-strain anti-influenza antibody production.

**Conclusion:**

We propose that non-infectious viral particles can be surface-engineered to produce antigen-presenting particles that mimic antigen-presenting cells to induce immune responses in human peripheral blood lymphocytes. The viral particles behave as "biological carriers" for recombinant proteins, thereby establishing a new therapeutic paradigm for molecular medicine.

## Background

While drug advances continue to be made in infectious disease and cancer biology, there remains an urgent need for the identification of new immunological approaches to address the problems of drug resistance, toxicity, and pharmacokinetic drug interactions [1-3]. Cell-based approaches in T cell expansion, adoptive transfer of lymphokine-activated killer cells, tumor infiltrating lymphocytes, and dendritic cell mediated antigen presentation have shown promise [4-9], but the broad application of these therapies are hampered due to difficulties in isolating and expanding appropriate cell populations and establishing the necessary cellular expansion to meet dosage requirements. Targeting strategies for *in vivo *gene therapy have also proven difficult [10], resulting in infection of non-targeted cell types and expression levels that are either inadequate or lead to uncontrolled adverse and problematic outcomes [11,12]. Genetic engineering of immune cells has the advantage of providing multiple epitopes and continuous antigen production [13], but in practice is too cumbersome to implement. In order to meet present and future clinical demands, a simpler approach is needed, one in which immune responses can be induced *in vivo *without the need for cellular engraftment and/or viral infection to deliver the therapeutic.

Advances in our understanding of cellular signal transduction in human physiology, suggests that stimulating cellular processes by cell surface engagement is possible. Accessory costimulatory molecules as represented in the B7- and TNF-family of proteins [14] are effective in vaccination studies [15,16]. Engineering biological vehicles that deliver intact costimulatory proteins instead of their genes may be more feasible and amenable to therapeutic immune modulation. There is a large body of literature showing that surface-engineering of viral particles occurs naturally as viruses are released from host cells [17-23]. Clearly, technology that mimics cellular antigen presenting properties by displaying the appropriate peptides required for T cell activation in the presence of costimuatory molecules while maintaining specificity would greatly facilitate infectious disease and tumor biology vaccine development.

Experiments are conducted in this report, to test if the properties of genetically engineered cells can be transferred to non-infectious viral particles with the hypothesis that antigen-presenting particles can replace antigen-presenting cells. To test this hypothesis, viral particles released from genetically-modified cells expressing costimuatory molecules are inactivated and added to human peripheral blood lymphocytes (PBL) cultures. Surface-engineered particles are compared to non-engineered particles and tested for their ability to stimulate T cell proliferation. The preparations are inactivated to eliminate cellular infection and to promote cell surface interactions. We report here the use of such particles in infectious diseases – human immunodeficiency virus type 1 (HIV-1), human simplex virus (HSV), and Influenza. Results suggests that viral particles derived from costimuatory expressing genetically-modified host cells can mimic mature antigen-presenting dendritic cells and are capable of activating T cell proliferation. We illustrate that virion particles derived from host cells expressing costimuatory molecule on their surface can induce immune responses that are specific to and dependent on the virus used to create the particle.

## Results

### Non-infectious particles derived from antiCD3- and B7-engineered host cells can stimulate human PBL proliferation

The original observation that magnetic-bead bound CD3 and CD28 antibodies prevent monocytotropic HIV-1 infection of peripheral blood CD4-positive T cells [24] spawned two approaches that were experimentally tested. In the first approach, human mesenchymal stem cells were engineered to express the costimuatory molecules CD80/B7.1 and CD86/B7.2, the natural ligands for the T cell CD28 receptor, and fragment C of tetanus toxoid. Implantation of these cells in mice resulted in successful *in vivo *induction of tetanus toxoid specific immune responses [25]. Although successful, the approach is still not amendable to large-scale production and distribution due to cellular expansion requirements. For this reason, the implantation of gene-engineered human mesenchymal stem cells show little advantage over the original CD4-positive T cell expansion approach; both approaches require cellular expansion and without an amplification of the therapeutic moiety, the potential large-scale medical benefits of these cell-based approaches are limited.

In the second (current) approach we constructed cell lines expressing costimuatory molecules on their surface. Once established, the cells were virally infected and the released virus collected, inactivated, and tested for their ability to activate T cells. Our hypothesis is that the viral particles released from appropriately engineered cells would attain the T cell activation potential of the host cells. If successful, therapeutic moieties expressed on a cell's surface could be transferred and presented on the surface of released viral particles. By producing engineered particles with properties similar to the engineered cells, viruses released from these cells amplify the therapeutic moiety many fold since each cell expresses 10^3 ^to 10^9 ^virus particles. By this procedure, each virus is surface-engineered, bestowing antigen-presenting properties to the released particles. We tested this approach with viral-infected cells expressing antiCD3 single-chain antibody and CD80/CD86 costimuatory molecules.

**Figure 1:**
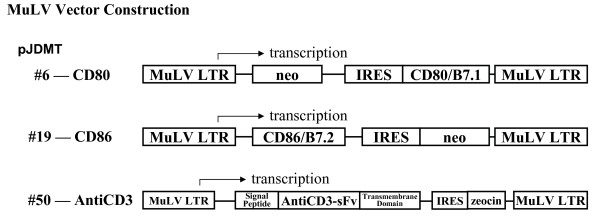
Schematic representation of the retroviral vector constructions used to surface-modify particle-producing host cell lines. The detail construction of the vectors used in this report, pJDMT#6 (CD80/B7.1), pJDMT#19 (CD86/B7.2), and pJDMT#50 (antiCD3-sFv) are described in the Materials and Methods section.

The first step in surface-engineered virion production is the establishment of host cell lines expressing the therapeutic molecules. We genetically-modified host cell lines using retroviral vector constructions (Fig. 1) to permanently express antiCD3 single-chain antibody and the natural ligands for the CD28 T cell receptor, CD80/B7.1 and CD86/B7.2, on the host cell surface. Three sets of cell lines were established based on: Lof(11-10) cells [26]; 1119, a chronic HIV-expressing cell line; and Madin-Darby canine kidney (MDCK) cells [27]. These cell lines are the host cells for the production of surface-engineered HSV-2, HIV-1, influenza-A, and influenza-B particles, respectively. Each modified cell line was tested in co-culture experiments with human PBLs to demonstrate that the cells themselves could induce T cell proliferation (data not shown). The Lof(11-10) and MDCK cells were infected with HSV-2 and influenza-A/-B viruses, respectively; the 1119 cell line was induced to synchronically express HIV-1. Particles were collected from viral-infected modified cells and compared to control particles expressed from non-modified viral-infected cells. The particle preparations were inactivated by treatment with the DNA cross-linking agent, aminomethyltrimethyl psoralen (AMT) followed by ultraviolet irradiation.

**Figure 2:**
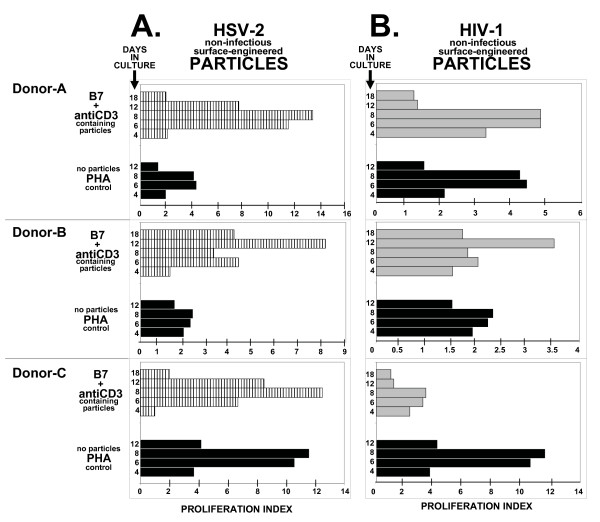
Comparison of proliferation index (PI) in three donors (A, B, and C) human PBLs cultured with either PHA or particles surface-engineered with CD80, CD86, and antiCD3-sFv (B7+antiCD3). In **Panel A**, surface-engineered HSV-based particles are derived from HSV-2 infected genetically surface-modified Lof(11-10) cells (horizontal hatched bars). In **Panel B**, surface-engineered HIV-based particles are derived from genetically surface-modified 1119 cells that are chronically-expressing human immunodeficiency virus type-1 (gray-filled bars). The time course shown is 4, 6, 8, and 12 days for PHA-treated cultures; 4, 6, 8, 12, and 18 days for surface-engineered particle treated cultures. Proliferation Index establishes a proliferation ratio between exposed cultures and non-exposed cultures. PHA treated cultures are not exposed to particles. For PHA (black-filled bars), the proliferation value in the presence of PHA (i.e. Donor-A, 6 hour timepoint = 10,900 relative fluorescent units) is divided by untreated cultures not exposed to PHA (i.e. Donor-A, 6 hour timepoint = 2,500 relative fluorescent units); for B7+antiCD3, the proliferation value in the presence of B7+antiCD3 surface-engineered particles (i.e. Donor-A, 6 hour timepoint = 26,700 relative fluorescent units for HSV-2 in panel A; 11,000 relative fluorescent units for HIV-1 in panel B) is divided by the proliferation value observed with non-engineered viral-based particles (i.e. Donor-A, 6 hour timepoint = 2,300 relative fluorescent units for HSV-2 in panel A; 2,300 relative fluorescent units for HIV-1 in panel B). The remaining PI values are calculated in a similar fashion. Almost identical "background" values are observed for non-PHA exposed and non-engineered particles in Donors-A, -B, and -C cultures. Actual induced values can be calculated by multiplying the PI value by the "background" value. Particle preparations used in this figure were PEG-concentrated (200× for HIV; 25× for HSV) and inactivated to render them non-infectious.

T cell proliferation assays illustrate the ability of non-infectious surface-engineered HSV-2 and HIV-1 particle preparations to stimulate human peripheral blood T cells obtained from healthy donors (Fig. 2A: HSV-2; Fig. 2B: HIV-1). Results from three separate donor's lymphocytes (Donors-A, -B, and -C) are shown for each test virus. The data shows the fold increase in T cell proliferation with particles derived from CD80/CD86 (B7) and antiCD3 single-chain antibody (B7+antiCD3) modified host cells relative to the degree of T cell proliferation with phytohemagglutinin (PHA) activation, where no particles were added. PHA treatment serves as a donor-specific standardization control for proliferation potential. In these experiments, the HSV-2 based engineered particles (Fig. 2A) stimulated T cell proliferation more than HIV-1 based engineered particles (Fig. 2B). The results show Proliferation Index (PI) values of 8 to 14 for HSV-based and PI values of 4 to 5 for HIV-based particles. These numbers compared to PI values of 2 to 12 in PHA stimulated cultures. With the exception of HIV-1 based particles in PBLs from Donor-C, engineered particles stimulated T cells as well as and in some cases better than PHA treatment. Although less than PHA treatment, HIV-1 based particles did induce Donor-C T cell proliferation with PI values of 1 to 4 over the time course measured.

**Figure 3:**
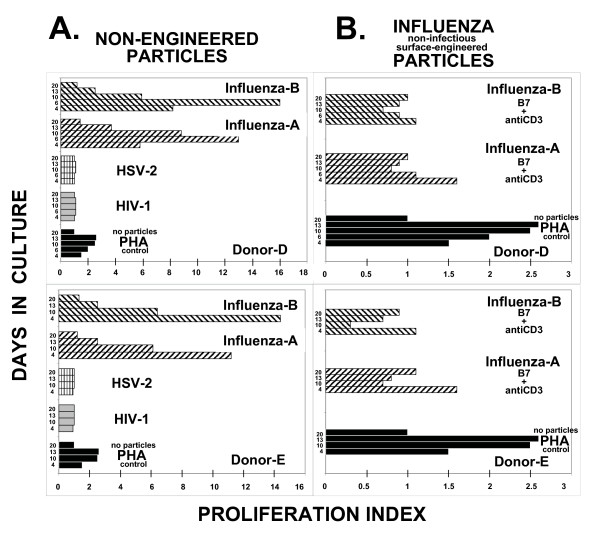
Proliferation Index (PI) time course comparison in two donor (D and E) PBLs. **Panel A: **Non-engineered particles. For PHA (black-filled bars), the proliferation value in the presence of PHA (i.e. Donor-D, 6 hour timepoint = 4,000 relative fluorescent units) is divided by the value observed in untreated cultures (i.e. Donor-D, 6 hour timepoint = 2,000 relative fluorescent units); for HIV-1 (gray-filled bars), the proliferation value in the presence of non-engineered HIV-1 particles (i.e. Donor-D, 6 hour timepoint = 2,200 relative fluorescent units) is divided by the value observed in untreated cultures (i.e. Donor-D, 6 hour timepoint = 2,000 relative fluorescent units); for HSV-2 (horizontal hatched bars), the proliferation value in the presence of non-engineered HSV-2 particles (i.e. Donor-D, 6 hour timepoint = 2,000 relative fluorescent units) is divided by the value observed in untreated cultures (i.e. Donor-D, 6 hour timepoint = 2,000 relative fluorescent units); for Influenza A (PR8) (right-diagonal hatched bars), the proliferation value in the presence of non-engineered influenza-A particles (i.e. Donor-D, 6 hour timepoint = 26,000 relative fluorescent units) is divided by the value observed in untreated cultures (i.e. Donor-D, 6 hour timepoint = 2,000 relative fluorescent units); and for Influenza B (Russian) (left-diagonal hatched bars), the proliferation value in the presence of non-engineered influenza-B particles (i.e. Donor-D, 6 hour timepoint = 32,000 relative fluorescent units) is divided by the value observed in untreated cultures (i.e. Donor-D, 6 hour timepoint = 2,000 relative fluorescent units). **Panel B: **Surface-engineered influenza particles. For B7+antiCD3 surface-engineered influenza A (PR8) (right-diagonal hatched bars), the proliferation value in the presence of surface-engineered particles (i.e. Donor-D, 6 hour timepoint = 29,000 relative fluorescent units) is divided by the proliferation value observed with non-engineered influenza A particles (i.e. Donor-D, 6 hour timepoint = 26,000 relative fluorescent units); and for Influenza B (Russian) (left-diagonal hatched bars), the proliferation value in the presence of surface-engineered particles (i.e. Donor-D, 6 hour timepoint = 28,800 relative fluorescent units) is divided by the proliferation value observed with non-engineered influenza B particles (i.e. Donor-D, 6 hour timepoint = 32,000 relative fluorescent units). The time course shown in panels A and B for Donor-D is 4, 6, 10, 13, and 20 days; for Donor-E is 4, 10, 13, and 20 days. The remaining PI values are calculated in a similar fashion. Actual induced values can be calculated by multiplying the PI value by the "background" value. Particle preparations used in this figure were PEG-concentrated (200× for HIV; 25× for HSV; 40× for Influenza A/B) and inactivated to render them non-infectious.

What is not obvious from the PI data is that the HSV-2 and HIV-1 non-engineered particles do not stimulate T cell proliferation; cells from two different donors (Donor-D and -E) treated with non-engineered particles gave PI values of 1, with no T cell proliferation ability (Fig. 3A). This is distinct from non-engineered particles formed from influenza-A and influenza-B viruses, where PI values as high as 16 are observed (Fig. 3A). The figure show results from two separate donor PBLs (Donors-D and -E) where the addition of non-engineered influenza-A (PR8) and influenza-B (Russian) viral preparations increased T cell proliferation to levels that are 4- to 5-fold higher than PHA-stimulated control cultures where no influenza particles are added. Surface-engineered (B7+antiCD3) influenza-based particles did not further increase T cell proliferation over non-engineered particles (Fig. 3B). Therefore at least for influenza, similar PI values are observed in the presence and absence of surface engineering.

In addition to proliferation assays, cytokine (IFN-γ, IL-10, and IL-4) expression analyses were measured in the culture media (Table 1). Surface-engineered HIV-1 particles were compared to non-engineered HIV-1 particles generated from non-modified host cells; PHA-stimulated cultures in the absence of particles were used as a donor cell standardized control. Whereas, IFN-γ values between 450 and 810 pg/ml are observed in unstimulated cultures and in cultures exposed to non-engineered HIV-based particles, IFN-γ value of greater than 2,000 pg/ml are observed in cultures exposed to surface-engineered HIV-1 particles. B7 and B7+antiCD3 engineered particles stimulated IFN-γ production similar to that observed in PHA-stimulated cultures.

**Table 1:** Cytokine Profile for HIV-based Particles

		**Particle Preparations**		**Time Points**	
**Cytokine**	**Culture Treatment**^1^	**Virus**	**Modification**	**6 Day**	**13 Day**
**IFN-γ pg/ml**	Unstimulated	no particles	NA^2^	810	ND^3^
	PHA-stimulated	no particles	NA	>2,000	ND
	Unstimulated	HIV-1	non-engineered	450	650
	Unstimulated	HIV-1	B7-engineered	>2,000	>2,000
	Unstimulated	HIV-1	B7+antiCD3	>2,000	>2,000

**IL-10 pg/ml**	Unstimulated	no particles	NA	50	ND
	PHA-stimulated	no particles	NA	150	ND
	Unstimulated	HIV-1	non-engineered	70	50
	Unstimulated	HIV-1	B7-engineered	40	25
	Unstimulated	HIV-1	B7+antiCD3	30	30

**IL-4 pg/ml**	Unstimulated	no particles	NA	<10	ND
	PHA-stimulated	no particles	NA	<10	ND
	Unstimulated	HIV-1	non-engineered	<10	<10
	Unstimulated	HIV-1	B7-engineered	<10	<10
	Unstimulated	HIV-1	B7+antiCD3	<10	<10

^1^Data from Donor-K cells

^2^NA: not applicable

^3^ND: not done

However, unlike IFN-γ, the expression of IL-10 did not increase in cultures exposed to engineered HIV-1 particles, and in fact showed a slight decrease below the values observed in unstimulated control cultures (Table 1). A constitutive value of 50 and 70 pg/ml is observed in unstimulated culture and cultures exposed to non-engineered particles. Cultures exposed to either B7 or B7+antiCD3 surface-engineered particles showed 2- to 3-fold reduction in IL-10 values to between 25 and 40 pg/ml. No IL-4 was detected in any of the cultures tested (Table 1). At least for HIV, the procedure induces T helper (Th) type 1 (Th1) responses while reducing Th type 2 (Th2) responses.

### Surface-engineered particles with only the B7 costimulatory molecules can stimulate human PBL T cell proliferation

**Figure 4:**
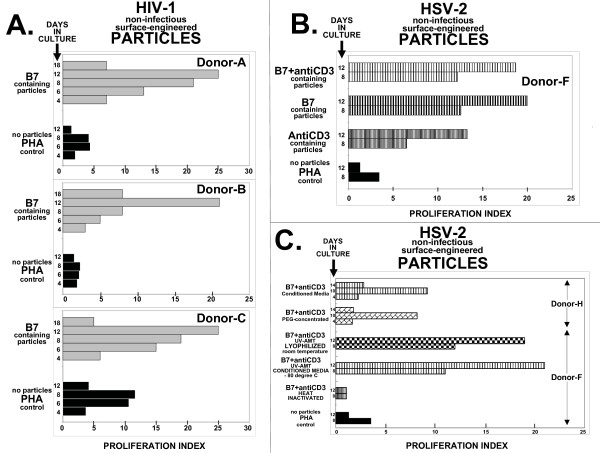
**Panel A: **Proliferation Index (PI) time course comparison in three donors' (A, B, and C) PBLs cultured with either PHA or B7 surface-engineered HIV-1 based particles. For PHA (black-filled bars), the proliferation value in the presence of PHA (i.e. Donor-A, 6 hour timepoint = 10,900 relative fluorescent units) is divided by untreated cultures not exposed to PHA (i.e. Donor-A, 6 hour timepoint = 2,500 relative fluorescent units); for B7 (gray-filled bars) the proliferation value in the presence of B7 surface-engineered particles (i.e. Donor-A, 6 hour timepoint = 32,500 relative fluorescent units) is divided by the proliferation value observed with non-engineered HIV-based particles (i.e. Donor-A, 6 hour timepoint = 2,500 relative fluorescent). The time course shown is 4, 6, 8, 12, and 18 days for B7; 4, 6, 8, and 12 days for PHA. Particle preparations used in this panel were PEG-concentrated (200× for HIV; 25× for HSV) and inactivated to render them non-infectious. **Panel B: **Proliferation Index (PI) time course comparison in Donor-F PBLs cultured with HSV-2 based surface-engineered particles. For PHA (black-filled bars), the proliferation value in the presence of PHA (i.e. 8 hour timepoint = 9,000 relative fluorescent units) is divided by cultures not exposed to PHA (i.e. 8 hour timepoint = 2,600 relative fluorescent units); for AntiCD3 surface-engineered particles (tightly packed horizontal hatched gray bars), the proliferation value in the presence of AntiCD3 (i.e. 8 hour timepoint = 15,000 relative fluorescent units) is divided by the proliferation value observed with non-engineered HSV-based particles (i.e. 8 hour timepoint = 2,300 relative fluorescent units); for B7 surface-engineered particles (horizontal hatched gray bars), the proliferation value in the presence of B7 (i.e. 8 hour timepoint = 29,000 relative fluorescent units) is divided by the proliferation value observed with non-engineered HSV-based particles (i.e. 8 hour timepoint = 2,300 relative fluorescent units); for B7+antiCD3 surface-engineered particles (horizontal hatched bars), the proliferation value in the presence of B7+antiCD3 (i.e. 8 hour timepoint = 28,000 relative fluorescent units) is divided by the proliferation value observed with non-engineered HSV-based particles (i.e. 8 hour timepoint = 2,300 relative fluorescent units). Particle preparations used in this panel were from conditioned media and inactivated to render them non-infectious. **Panel C: **Proliferation Index (PI) time course comparison in Donor-F PBLs cultured with HSV-2 based surface-engineered particles. For PHA (black-filled bars), the proliferation value in the presence of PHA (i.e. 8 hour timepoint = 4,200 relative fluorescent units) is divided by cultures not exposed to PHA (i.e. 8 hour timepoint = 1,200 relative fluorescent units); for Heat-Inactivated B7+antiCD3 surface-engineered particles (tightly packed horizontal hatched gray lines), the proliferation value in the presence of heat-inactivated surface-engineered particles (i.e. 8 hour timepoint = 7,300 relative fluorescent units) is divided by the proliferation value observed with heat-inactivated non-engineered HSV-based particles (i.e. 8 hour timepoint = 6,500 relative fluorescent units); for Conditioned Media B7+antiCD3 (horizontal hatched bars), the proliferation value in the presence of conditioned media from surface-engineered particles (i.e. 8 hour timepoint = 30,000 relative fluorescent units) is divided by the proliferation value observed in conditioned media from non-engineered HSV-based particles (i.e. 8 hour timepoint = 2,700 relative fluorescent units); for Lyophilized room temperature stored B7+antiCD3 (checker bars), the proliferation value in the presence of the lyophilized surface-engineered particles (i.e. 8 hour timepoint = 28,000 relative fluorescent units) is divided by the proliferation value observed with lyophilized non-engineered HSV-based particles (i.e. 8 hour timepoint = 2,300 relative fluorescent units). The above data was obtained using Donor-F PBLs. PEG-concentrated B7+antiCD3 (brick bars) proliferation value was compared to Conditioned Media B7+antiCD3 (horizontal hatched bars) in Donor-H PBLs. The remaining PI values are calculated in a similar fashion. Almost identical "background" values are observed for non-PHA exposed and non-engineered particles in Donors-A, -B, -C, -F, and -H cultures. Actual induced values can be calculated by multiplying the PI value by the "background" value. Particle preparations used in this panel unless otherwise identified were from conditioned media and inactivated to render them non-infectious.

In addition to B7+antiCD3 surface-engineered particle preparations derived from the three infectious agents (HIV-1, HSV-2, and Influenza), individual antiCD3 and CD80/CD86 (B7) costimulatory engineered particle preparations were also produced and tested. Initially, experiments were performed with these preparations to demonstrate the need for particles to contain both signals for T cell proliferation; the antiCD3 single-chain antibody molecule delivering signal one to the T cell receptor complex and B7 molecules delivering signal two to the CD28 receptor [15,24]. However to our surprise, the dual requirement was not needed for HSV-2 and HIV-1 based particle mediated T cell proliferation induction. Surface-engineered particles containing B7 alone (Fig. 4A: HIV-1; Donor-A, -B and -C) or AntiCD3 alone (Fig. 4B: HSV-2; Donor-F) are effective in stimulating T cell proliferation in human PBL cultures. The data shows that for HSV-2 based particles, a similar degree of T cell proliferation (PI = 20) was observed with B7+antiCD3 and B7 alone (Fig. 4B). However, HIV-1 based surface-engineered particles with B7 alone (Fig. 4A) displayed a more potent *in vitro *proliferation response than B7+antiCD3 engineered particles (Fig. 2B) – PI values of 20 to 25 for B7 particles, compared to PI values of 8 to 14 for B7+antiCD3 particles.

### Concentrate and room temperature storage of surface-engineered particles without loss of activity

Initial T cell proliferation experiments used conditioned media from surface-modified host cells. In order to partially purify and concentrate viral particle preparations, the traditional method of ultracentrifugation was considered, but due to its expensive, limited volume processing ability, and the potential removal of key surface components from the final product, we chose to use polyethylene glycol (PEG)-precipitation. PEG-precipitation has long been used to concentrate viral particles from serum samples and the procedure circumvents many of the drawbacks posed by ultracentrifugation and was the method of choice to concentrate surface-engineered particles. Culture media containing viral particles were harvested, clarified, PEG-precipitated, and compared biologically. These comparisons illustrate that the surface-engineered viral particles could be PEG-concentrated and still retain their ability to stimulate T cell proliferation (see Fig. 4C: Donor-H).

Since the particles are viewed as a scaffold that carries and maintains the orientation and conformation of the over-expressed host cell surface proteins, the technology does not require the particles to be infectious. The ability to use non-infectious particles as a biologic raises the possibility of storing the surface-engineered particles at room temperature as a lyophilized concentrate. To test this, conditioned media from B7+antiCD3 surface-modified host cells was compared to the same conditioned media that was lyophilized and stored for 3 weeks at room temperature for their ability to stimulate T cell proliferation. The results show that exposure of PBLs to either preparation result in almost identical PI values at 8 and 12 days (Fig. 4C: Donor-F). In addition, the figure demonstrates that heat treatment completely destroys the preparation's ability to stimulate T cell proliferation (Fig. 4C: Donor-F). The results support the conclusion that surface-engineered viral particles can be lyophilized, stored at room temperature, and still retain their ability to stimulate T cell proliferation.

### Functional assays illustrating HSV-2 and HIV-1 surface-engineered particle viral specificity

The data to this point suggests a non-specific ability of HSV-2 and HIV-1 surface-engineered particles to stimulate T cell proliferation. In order to determine if viral specificity exist between these particle preparations, we tested two functional assays to elucidate differences. The assays compared the ability of the particles to (1) inhibit HIV replication and (2) to induce specific antibody responses.

#### HIV replication inhibition

**Figure 5:**
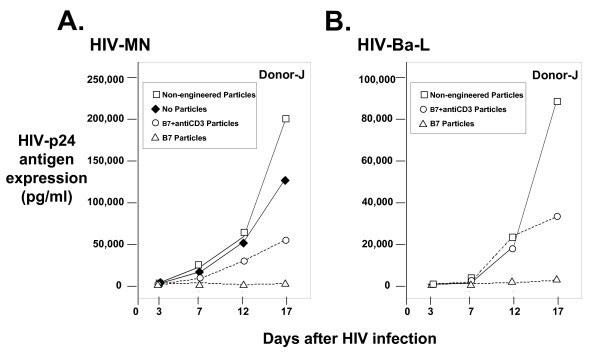
Surface-engineered HIV-based particle dependent inhibition of HIV-1 replication. **Panel A: **Lymphocytotropic HIV-1 MN p24 antigen expression in PHA-stimulated PBLs. **Panel B: **Monocytotropic HIV-1 Ba-L p24 antigen expression in PHA-stimulated PBLs. Donor-J cells were PHA-treated and exposed to either no particles (filled diamonds), non-engineered HIV-based particles (open squares), B7+antiCD3 surface-engineered HIV-based particles (open circles), or B7 surface-engineered HIV-based particles (open triangles). At day 3, cultures are washed and infectious HIV-1 is added – HIV-MN in Panel A and HIV-Ba-L in Panel B. Aliquots are removed at 3, 7, 12, and 17 days and HIV-1 encoded p24 antigen expression is determined by ELISA. Particle preparations used in this figure were PEG-concentrated and inactivated to render them non-infectious.

Experiments were design to test the ability of surface-engineered particles to inhibit HIV replication. Cultures of PBLs were PHA-stimulated to insure the ability of HIV to replicate. In addition, some cultures were also treated with either non-engineered or surface-engineered HIV-1 and HSV-2 based particles. After 3 days of stimulation and extensive washing of the cells to remove unbound material, the cells were resuspended in fresh media containing infectious HIV-1. Both monocytotropic (Ba-L and ADA) and lymphocytotropic (MN and HXB2) infectious HIV-1 preparations were used. Exposure of cultures to non-engineered particles (Fig. 5A and 5B, open squares) replicated HIV-1 to levels similar to control cultures where no particles were added (Fig. 5A, closed diamonds). The level of replication was monitored by p24 antigen released into the culture supernatants and robust amounts of p24 antigen were detected over the 17 day time period. Stimulation of cultures with PHA and exposure to HIV-based surface-engineered particles with either B7 (Fig. 5A and 5B, open triangles) or B7+antiCD3 (Fig. 5A and 5B, open circles) inhibited HIV-MN and HIV-Ba-L replication 86 and 90% or 59 and 88% respectively, in Donor-J cells (Table 2: Expt. 4). Similar inhibition is observed in other donors' PBLs. In donor M PBLs, an Inhibition Index of 55 and 71% (for B7 particles) or 95 and 94% (for B7+antiCD3 particles) were observed (Table 2: Expt. 1). Table 2 tabulates the results from four additional experiments (Expt. 2, 3, 5, and 6), with three different donor (N, O, and P) PBLs. An Inhibition Index value, which is the average inhibition value for all experimental time points, is used to summarize the percent inhibition results. For non-engineered particles, the percent inhibition was calculated at each time point by dividing the HIV-p24 antigen value observed in non-engineered particle cultures, to those where no particles were added; for B7 and B7+antiCD3 engineered particles, the percent inhibition was calculated at each time point by dividing the HIV-p24 antigen value observed in B7 and B7+antiCD3 supplemented cultures to those where no particles were added. In most cases, B7 surface-engineered particles inhibited HIV replication, better than B7+antiCD3 surface-engineered particles (Table 2).

**Table 2:** Percent Inhibition of HIV Replication

			**Particle Preparations**							
**Expt.**	**Infecting Virus**	**Inhibition Index**	**Virus**	**Modification**	**Time Points (days)**					**Donor**
					**6**	**14**				

**1.**	HIV-MN	**0**	HIV-1	non-engineered	0	0				M
		**55**		B7-engineered	70	40				
		**95**		B7+antiCD3	94	95				
		**0**	HSV-2	B7+antiCD3	0	0				
	HIV-Ba-L	**0**	HIV-1	non-engineered	0	0				
		**71**		B7-engineered	82	60				
		**94**		B7+antiCD3	90	98				
		**0**	HSV-2	B7+antiCD3	0	0				

					**6**	**17**				

**2.**	HIV-MN	**80**	HIV-1	B7-engineered	70	89				N
		**88**		B7+antiCD3	88	89				
		**0**	HHV-8	B7+antiCD3	0	0				

					**4**	**11**	**14**			

**3.**	HIV-MN	**96**	HIV-1	B7-engineered	92	98	99			O
		**55**		B7+antiCD3	56	45	63			

					**3**	**7**	**12**	**17**		

**4.**	HIV-MN	**0**	HIV-1	non-engineered	0	0	0	0		J
		**86**		B7-engineered	55	91	99	99		
		**59**		B7+antiCD3	46	67	51	73		
	HIV-Ba-L	**0**	HIV-1	non-engineered	0	0	0	0		
		**90**		B7-engineered	60	98	100	100		
		**88**		B7+antiCD3	72	97	99	84		

					**4**	**6**	**9**	**12**		

**5.**	HIV-MN	**89**	HIV-1	B7-engineered	65	97	98	97		P
		**75**		B7+antiCD3	73	96	82	48		

					**4**	**6**	**9**	**12**	**17**	

**6.**	HIV-MN moi = 1	**85**	HIV-1	B7-engineered	57	80	96	96	94	P
		**76**		B7+antiCD3	59	82	90	90	59	
	HIV-MN moi = 2	**74**	HIV-1	B7-engineered	33	95	75	75	94	
		**79**		B7+antiCD3	40	92	85	85	95	
	HIV-MN moi = 4	**52**	HIV-1	B7-engineered	55	97	35	35	40	
		**38**		B7+antiCD3	60	54	36	36	2	
	HIV-MN moi = 8	**40**	HIV-1	B7-engineered	53	60	0	0	8	
		**28**		B7+antiCD3	37	60	20	20	2	

In addition to demonstrating that non-infectious surface-engineered HIV-1 particles inhibit HIV replication, the data also illustrates that neither surface-engineered HSV-2 (Table 2: Expt. 1), nor engineered human herpesvirus type-8 (HHV-8) particles (Table 2: Expt. 2) were able to inhibit HIV replication. The addition of similarly engineered heterologous viral particles did not inhibit HIV replication; inhibition of HIV replication required both surface modification and the HIV virion. These experiments demonstrate biological differences between the engineered particle preparations, where only HIV-based particles inhibit HIV replication.

Two independent sets of experiments were conducted to demonstrate that the inhibition of HIV replication was not due to depletion and/or apoptosis of CD4-positive cells. In the first set of experiments, the amount of infectious virus was increased two-, four-, and eight-fold higher and the ability of a constant amount of engineered particles to inhibit HIV replication was monitored (Table 2, Expt.-6). Results from these experiments show that the degree of inhibition is reduced as the amount of HIV inoculum is increased. For B7 engineered particles, the Inhibition Index changed from 85% (moi = 1), to 74% (moi = 2), to 52% (moi = 4), to 40% (moi = 8); and for B7+antiCD3 engineered particles, the inhibition index changed from 76% (moi = 1), to 79% (moi = 2), to 38% (moi = 4), to 28% (moi = 8). Thus, the degree of HIV-inhibition mediated by surface-engineered HIV particles is reduced as the amount of viral inoculum increases; the observed inhibition is titratable.

In addition to the biological infectivity assay to illustrate the presence of CD4-positive cells, the CD4/CD8 ratio in treated cultures was monitored by flow cytometry (Table 3). Unstimulated and PHA/IL-2 stimulated T cells were compared to T cells treated with HIV-based particles in the presence and absence of infectious HIV exposure. Cultures treated with non-engineered particles show similar CD4 and CD8 cell percentages, ratios, and mean fluorescence values as no particle treated cultures. CD4 percentages of 51 versus 55 with mean fluorescence of 1400 and 1100 were observed; CD8 percentages of 38 were seen for both with mean fluorescence intensity of 660 and 580 for no particle treated and non-engineered particle treated cultures, respectively [Table 3: 1(a)]. Unstimulated cultures treated with particles derived from either B7 or B7+antiCD3 surface-engineered HIV particles, exhibited a decrease in the percentage and mean fluorescence of CD4 positive cells [Table 3: 1(a)]. The decrease in CD4 cell percentage and mean fluorescence intensity was also observed in parallel PHA/IL-2 treated cultures with surface-engineered particle addition [Table 3: 1(b)]. If this observed decrease in day-3 cultures are due to "masking" of CD4 epitopes by the surface-engineered HIV particles is presently unknown, but upon removal of these particle from the culture at day-3 and further incubation, 8-day non-infected B7+antiCD3 cultures showed a 15% increase in the percentage of CD4 (63%) compared to either no particle treated (46%) or non-engineered particle treated (47%) cultures with similar mean fluorescence intensity [Table 3: 2(a)]. The CD4 cell percentage increase is maintained in cultures in the presence of either HIV-MN [Table 3: 2(b)] or HIV-Ba-L [Table 3: 2(c)] infection. The increase in the percentage of CD4 is accompanied with a decrease in the percentage of CD8 positive cells. Overall, the flow cytometry results together with the enhanced HIV replication as the infectious inoculum is increased in the biological infectivity assay, supports the premise that the observed inhibition of HIV replication is not due to massive apoptosis of CD4-positive cells.

**Table 3:** Flow Cytometry Analysis of Particle-treated Cultures

			**Particle Preparation**		**Percent Positive Cells** **(Mean Fluorescence Intensity)**		
	**Infecting Virus**	**Culture Treatment**^1^	**Virus**	**Modification**	**CD4**	**CD8**	**CD4/8**
**1. Time Point: 3 Days**							

**(a)**	None	None	None	None	51 (1400)	38 (660)	1.3
			HIV-1	non-engineered	55 (1100)	38 (580)	1.4
			HIV-1	B7-engineered	48 (70)	34 (390)	1.4
			HIV-1	B7+antiCD3	43 (90)	38 (460)	1.1
**(b)**	None	PHA/IL-2	None	None	50 (780)	47 (470)	1.1
			HIV-1	non-engineered	54 (560)	39 (360)	1.4
			HIV-1	B7-engineered	37 (95)	45 (250)	0.8
			HIV-1	B7+antiCD3	38 (100)	34 (230)	1.1

**2. Time Point: 8 Days**							

**(a)**	None	PHA/IL-2	None	None	46 (1600)	55 (370)	0.8
			HIV-1	non-engineered	47 (1300)	51 (270)	0.9
			HIV-1	B7+antiCD3	63 (1100)	38 (220)	1.6
**(b)**	HIV-MN	PHA/IL-2	None	None	40 (1400)	59 (360)	0.7
			HIV-1	non-engineered	41 (1360)	61 (310)	0.7
			HIV-1	B7+antiCD3	47 (1260)	58 (280)	0.8
**(c)**	HIV-Ba-L	PHA/IL-2	None	None	35 (1320)	61 (410)	0.6
			HIV-1	non-engineered	45 (1460)	59 (340)	0.8
			HIV-1	B7+antiCD3	49 (1100)	54 (225)	0.9

^1^Data from Donor-L cells

#### Induction of specific antibody responses

Unlike HIV-1, HSV-2 does not replicate in PBLs and replication inhibition assays could not be done. In order to demonstrate HSV specific responses, humoral immune response experiments were performed (Table 4, Expt. 1 and 2).

**Table 4:** Particle-induced Antibody Formation

	**Particle Preparations**										
**Expt.**	**Virus**	**Modification**	**Relative Optical Density Units**								
**1.**			**HSV-1 Coated**			**HSV-2 Coated**			**HIV-1 Coated**		
			
			**9D**	**13D**	**16D**	**9D**	**13D**	**16D**	**9, 13, 16 Days**		
			
	None	None	0.089	0.034	0.02	0.006	0.006	0.005	0.006		
	HSV-2	non-engineered	0.050	0.019	0.010	0.006	0.006	0.005	0.005		
		B7-engineered	**3.6**	**16.5**	**7.9**	**0.2**	**0.3**	**0.3**	0.004		
		B7+antiCD3	**3.7**	**3.7**	**6.8**	**0.3**	**0.4**	**0.7**	0.005		

**2.**			**HSV-1 Coated**			**HSV-2 Coated**			**HIV-1 or VSV Coated**		
			
			**3D**	**6D**	**10D**	**3D**	**6D**	**10D**	**3D**	**6D**	**10D**
	None	None	0.007	0.005	0.005	0.005	0.005	0.005	0.005	0.005	0.063
		PHA/IL-2	0.005	0.012	**0.7**	0.005	0.004	**0.1**	0.007	0.007	0.005
	HSV-2	non-engineered	0.002	**1.4**	**5.2**	0.008	**1.3**	**0.8**	0.003	0.003	0.001
		antiCD3	**0.2**	**1.9**	**10.0**	**0.2**	**1.7**	**3.0**	0.003	0.004	0.005
		B7-engineered	0.001	**0.9**	**7.5**	0.003	**0.7**	**1.5**	0.003	0.008	0.004
		B7+antiCD3	0.001	**1.3**	**6.5**	0.025	**1.2**	**7.3**	0,004	0.005	0.004
	HIV-1	non-engineered	0.002	0.006	0.006	0.004	0.019	0.004	0.004	0.004	0.004
		B7-engineered	0.001	0.008	0.023	0.004	0.004	0.021	0.004	0.003	0.002
		B7+antiCD3	0.008	0.007	0.031	0.004	0.004	0.027	0.006	0.001	0.005

**3.**			**Influenza-A Japan Coated**			**Influenza-B Taiwan Coated**			**Influenza-B Russian Coated**		
			
			**11D**	**15D**		**11D**	**15D**		**11D**	**15D**	
			
	None	None	0.016	0.030		0.005	0.037		0.006	0.007	
		PHA/IL-2	0.16	0.030		0.010	0.046		0.018	0.007	
	Influenza-A	non-engineered	0.010	0.005		0.010	0.046		0.018	0.007	
	Japan	antiCD3	0.014	0.004		**0.22**	**0.29**		**0.21**	**0.15**	
		B7-engineered	0.011	0.004		0.008	0.006		0.014	0.005	
		B7+antiCD3	0.012	0.006		0.009	0.011		0.023	0.006	
	Influenza-A	non-engineered	0.013	0.004		0.006	0.005		0.003	0.004	
	PR8	antiCD3	0.009	0.004		0.010	0.008		**0.15**	**0.16**	
		B7-engineered	**0.40**	**0.34**		0.004	0.004		0.017	0.011	
		B7+antiCD3	**0.10**	0.031		0.008	0.004		0.009	0.003	
	Influenza-B	non-engineered	0.013	0.004		0.004	0.004		0.003	0.005	
	Taiwan	antiCD3	0.010	0.007		0.008	0.005		0.007	0.004	
		B7-engineered	**0.16**	**0.09**		0.005	0.004		0.004	0.004	
		B7+antiCD3	0.011	0.002		0.005	0.005		0.007	0.003	

Cultures of unstimulated PBLs were exposed to different viral-based AntiCD3, B7, and B7+antiCD3 engineered particles; aliquots were removed at the time points indicated and placed in 96-well plates that were coated with various lysed whole virus preparations. Wells were coated with lysates (detergent disrupted virions) obtained from purified preparations of HSV-1, HSV-2, HIV-1, vesicular stomatitis virus (VSV), and/or host cells (not shown). An aliquot of cells from each culture was placed into the various viral antigen-coated wells and incubated for 3-days. The cells were then removed, the wells washed, and the viral antigen coated wells were assayed for the presence of human antibodies. Detection was accomplished by monitoring the binding of horseradish peroxidase conjugated human antibody to each well. The ability of surface-engineered viral particles to produce human antibodies against the viral antigens was compared to cultures treated with non-engineered viral particles and cultures not treated with any particle preparation.

Treatment of PBL cultures with non-engineered HSV-2 particles and no particle treated cultures showed neither HSV-1 nor HSV-2 specific antibody formation (Table 4-1). However, cultures incubated with B7 and B7+antiCD3 surface-engineered HSV-2 based particles induced both HSV-1 and HSV-2 antibody formation at 9, 13, and 16 days (Table 4-1). The specificity of the HSV-2 engineered particle response was demonstrated in that no human antibody formation is observed on HIV-1 coated plates (Table 4-1). Experimental results shown in Table 4-2 illustrate that in some donor cells, PHA/IL-2 stimulated and unstimulated non-engineered HSV-2 based particles can also induce HSV antibody responses. However in all donor cells tested, HSV based particles did not induce non-HSV antibody responses; HSV-2 particles could not induce antibody responses to either HIV-1 or VSV (Table 4-2). In addition, HIV engineered particles did not induce antibody responses to any of the tested antigens.

### Functional assays illustrating surface-engineered Influenza-based particle induction of cross-strain antibody formation

We tested the ability of influenza-based particles to induce influenza-specific antibody responses. Using assays similar to those described for detecting HSV-2 specific antibody induction, PBLs exposed to surface-engineered influenza particles were incubated on viral-antigen coated plates. The detection of human antibodies using a conjugated horseradish peroxidase antibody was used to detect human influenza antibody production. Cultures of unstimulated PBLs were exposed to PEG-concentrated Influenza-A (Japan), Influenza-A (PR8), and Influenza-B (Taiwan) particles; aliquots were removed at the time points indicated (11 and 15 days) and placed in 96-well plates that were coated with lysed whole virus preparations of Influenza-A (Japan), Influenza-B (Taiwan), and Influenza-B (Russian). After 3 days in culture, the cell-free panels were incubated with horseradish peroxidase conjugated human antibody and detection of a signal was indicative of human antibody production (Table 4, Expt. 3).

No Influenza specific antibody formation is observed in PBL cultures where no particles, PHA/IL-2, and non-engineered Influenza particles were added (Table 4, Expt. 3). However, cultures incubated with AntiCD3, B7, and B7+antiCD3 engineered Influenza-based particles induced Influenza-specific antibody responses (Table 4, Expt. 3). The AntiCD3 engineered Influenza-A (Japan) based particles induce immune responses against influenza-B (Taiwan) and influenza-B (Russian) strains. The AntiCD3 surface-engineered Influenza-A (PR8) based particles induced antibodies to the Influenza-B (Russian) strain; the B7 and B7+antiCD3 surface-engineered particles induced antibodies to Influenza-A (Japan), but not Influenza-B (Taiwan) or Influenza-B (Russian) strains. The B7 surface-engineered Influenza-B (Taiwan) based particles induce antibodies to Influenza-A (Japan) antigens, but not the Influenza-B (Russian) strain. Although sporadic and not inducing antibody responses to the same strain used to form the particles, the cross-strain inductive response is similar to that observed between HSV-1 and HSV-2 responses induced from surface-engineered HSV-2 based particles. What appears clear is that engineered influenza A particles induced influenza B immune responses and vice-versa.

## Discussion

In this report, we describe a generic process to generate surface-engineered particles and functionally illustrate their use to induce immune responses. By choosing costimuatory accessory proteins (CD80/B7.1, CD86/B7.2) and a single-chain antibody (CD3-scFv) as our test molecules, we illustrate a process to induce specific T cell responses against infectious agents. We show that responses are observed with particles released from cells expressing these surface molecules that are not shown with particles released from cells where these molecules are not present. From the comparison of particles released from modified and non-modified cells, we conclude that the particles released from surface-modified cells are in themselves modified or engineered with properties similar to the modified cells. In effect, each engineered particle functions as a modified host cell. The inactive viral particle provides the scaffold to carry the viral-specific processed peptides presented on host MHC molecules and the engineered costimulatory (CD80/B7.1, CD86/B7.2) and/or antibody (CD3-scFv) molecules. Since the active moiety is presented on the viral particle surface, engagement with its cognate receptor induces cellular signal transduction pathways. Neither infectivity nor integration is required and the particles can be inactivated and lyophilized while still retaining their ability to induce signal transduction pathways and the resulting biological activity. The ability to amplify the therapeutic moiety by transferring surface molecules from cells to particles provides an economy-of-scale that could allow higher production of therapeutics at lower manufacturing cost, significantly enhancing the availability and introduction of biologic-based material for medical applications. By so doing, surface-engineered particles renew the intended promise of biotechnology for more selective drugs that are better tolerated and cheaper to make for large-scale production [37].

The cellular engineered molecules are incorporated into virions by the innate ability of viruses to incorporate host expressed surface proteins as they egress from their host cell. This phenomenon is intensively studies for HIV, ever since the first report [38] that beta-2 microglobulin and the alpha and beta chains of human lymphocyte antigen (HLA) DR were found present in sucrose density gradient-purified HIV and simian immunodeficiency virus preparations. We agree with the viewpoint of Tremblay et al. [39] that the mechanism responsible for HIV acquisition of host-encoded proteins is a passive inclusion model where the over-expression of specific molecules present in host cell membranes are incorporated into virion particles. Discrepancy exists in the literature on the ability of HIV to incorporate CD80 and CD86 into HIV virions [40,41], but the forced over-expression by retroviral transduction of these molecules and other molecules (CD3-scFv) onto the surface of virus-expressing host cells makes this point moot. Although host surface molecule incorporation is shown to occur for some members of the herpesvirus family – Epstein-Barr virus [42] and cytomegalovirus [43]; other RNA virus members – HTLV-1 [43,44]; various leukemia viruses [45-47]; and other DNA viruses – vaccinia [48], to our knowledge this report is the first to functionally demonstrate host surface molecules incorporation into HSV-2 and Influenza virions. In fact, the present report is the first to use the observation that host surface proteins are incorporated into virion particles as a potentially therapeutic modality. The present report is unlike any other in that the particles are surface-engineered and inactivated to make them non-infectious and potentially safe. Host cells are genetically-engineered to express costimuatory molecules and the particles are inactivated so that they behave as "biological carriers" of the over-expressed protein. This approach has universal applications in protein, antibody, and/or peptide *in vivo *delivery and may also be useful in directing viral cellular tropism in vector gene transfer applications [49] and nucleic acid delivery of small interfering RNAs. The approach, as applied here, is used to stimulate CD28-mediated signal transduction pathways (by incorporating B7-family members) and/or stimulation through the T cell receptor complex (by incorporating CD3-scFv antibody). The CD80/CD86 molecules are the natural ligands to the CD28 and CTLA-4 molecules on T cells; the CD3-scFv molecule displays a T cell activating single-chain antibody polypeptide derived from the antiCD3 OKT3 monoclonal antibody. By using the natural ligands of the CD28 molecule that requires additional signaling through MHC molecules for CD28-mediated signal transduction pathway activation, we avoid overall non-specific CD28 molecule activation that is detrimental to the host [50]. The interaction of OKT3-IgG and B7 molecules with the T cell receptor (TCR) and the CD28 receptor on T cells respectively lead to T cell proliferation [51]. In fact, surface-engineering virion particles with CD3-scFv molecule represent a novel approach to antibody production and manufacturing. Antibody manufacturing is complicated by the complexity of the technology, high costs, and long development times. The present technology illustrates the potential ease of production that could facilitate antibody application to diagnostic research [52] in addition to therapeutic applications.

We have compared surface-engineered particles to non-engineered particles (control particles) in three independent immune assays. The first is T cell proliferation assays, demonstrating that the surface engineered particles have intrinsic ability to stimulate T cells. In all cases, non-surface engineered particles derived from non-modified host cells are used as a control. The HSV-2 and HIV-1 control particles show no T cell proliferation ability; the control particles are derived from the same cells that are modified to create the surface-engineered particles, but without surface expression of the recombinant costimulatory molecules. Whereas resting PBLs express CD28; T cell activation induces CTLA-4 expression [51]. The differential regulation of CD28 and CTLA-4 on resting and activated cells may explain the observe differences in particle stimulation between CD80/CD86 surface-engineered particles and CD80/CD86/CD3-scFv surface-engineered particles (Fig. 4A and Fig. 2B, respectively). The HIV particles surface engineered with CD80/CD86 induced T cell PI values as high as 25, compare to PI values of 5 for those with CD80/CD86 and CD3-scFv addition. Possibly, the inclusion of CD3-scFv to the CD80/CD86 engineered HIV particles stimulates CTLA-4 expression on the T cell, dampening the degree of T cell proliferation due to the engagement of CD80/CD86 with the CTLA-4 molecule. However, the varying degrees of T cell proliferation between CD80/CD86 engineered particles with and without the addition of CD3-scFv is not observed in experiments with surface engineered HSV-2 particles (Fig. 4B).

The third immune assay monitored the ability of surface-engineered non-infectious viral particles to induce specific viral antibody responses. With the exception of HIV surface-engineered particles where no antibodies are detected, HSV and influenza surface-engineered particles induce viral-specific antibody production. Furthermore, the antibodies produced are cross-reactive – HSV-2 surface-engineered particles induce HSV-1 and HSV-2 specific antibodies (Table 4, Expt. 1 and 2); influenza A and B surface-engineered particles induce cross-strain influenza specific antibodies (Table 4, Expt.3). The ability of surface-modified influenza particles to induce cross-strain antibody formation may prove useful in flu vaccine applications. Instead of predicting with accuracy the flu strains circulating the globe in a given flu season, surface-modified Influenza particles could offer cross protection against other unexpected flu strains that may develop as the season progresses. Similarly, a surface-modified Influenza virion approach could prove instrumental in the development of an avian flu vaccine in future global flu pandemic [53]. Although enticing for influenza, the surface-modified influenza data is preliminary in nature and is complicated by the fact that the particles are produced in canine cells, the MDCK cell lines, and not in human cells; the degree of interaction between the canine MHC molecules and the human TCR complex is unknown. However, surface-modified influenza viral particles derived from modified human cells engineered to make influenza particles by reverse genetics methods [54-56] could produce more effective cross-strain antibodies. Possibly, surface-engineered particles derived from modified human host cells could prove useful in the production of cross-strain antibodies that are neutralizing in nature. Presently, there are no indications that any of the antibodies formed from surface-engineered HSV-2 or influenza particles are able to neutralize infectious virus.

The particle-based approach, unlike other virus-based delivery systems, can stimulate cells through signal-transduction independent of cellular activation; requires neither infection nor genetic incorporation; results in an amplification of the initial signal via intercellular pathways; and has targeted specificity in that only cells with the corresponding ligand in connection with MHC molecules are stimulated. The actual mechanism of action induced by the surface-engineered particles is not clear and appears to be dependent on the virus used to construct the particles.

## Conclusion

We functionally demonstrate the formation and use of non-infectious surface-engineered virion particles to induce immune responses against infectious diseases. The use of these particles is illustrated with three infectious agents and the technology is applicable to RNA as well as DNA viruses. In addition to potential immunotherapy applications, the technology can display any protein and/or single-chain antibody for use in other therapeutic and delivery application. The technology is based on the perfected art of virus release from host cells. The formation of surface-engineered virion particles during virus egress further attest to large-scale production and manufacturing capabilities of the therapeutic product. The technology lends itself to an off-the-shelf product for infectious disease and tumor biology immunotherapy and since these particles are purposefully engineered, these particles contribute to future nanotechnology initiatives.

## Materials and methods

### Host cell lines

Lof(11-10) cells are an SV40 T-antigen immortalized human hematopoietic stromal cell line [26]; Madin-Darby canine kidney (MDCK) cells are a canine cell line used for the *in vitro *growth of influenza viruses [27]; and 1119 cells are a chronic HIV-expressing human T cell line constitutively expressing intact low-titer (10^2 ^pfu/ml) virions with high-level (p24 > 0.1 mg/ml) defective particle formation. Lof(11-10) and MDCK cells are cultured in DMEM media, and 1119 cells are cultured in RPMI media. All media is supplemented with 10% heat-inactivated fetal calf serum.

### Viral strains

Twelve different viruses are used in the experiments outlined in this report and they can be divided into 3 groups: (i) For particle formation – HSV-2 strain G; Influenza A (Japan/305/57, H_2_N_2_); Influenza-A (PR/8/34, H_1_N_1_); Influenza B (Taiwan/2/62); and HHV-8 strain KS-1. (ii) For infectious HIV challenge assays – HIV-1 monocytotropic virus strain Ba-L and lymphocytotropic virus strain MN. (iii) For antibody detection – HSV-1 MacIntyre strain ; HSV-2 strain G; HIV-1 strain IIIB; vesicular stomatitis virus (VSV); Influenza-A (Japan/305/57, H_2_N_2_); Influenza-B (Taiwan/2/62) and Influenza-B (Russia/69). All viral preparations were obtained from Advanced Biotechnologies Incorporated (Columbia, MD).

### Establishing modified host cell lines: construction of retroviral vectors, retroviral production, and stable transduction of host cell lines

AntiCD3, B7, and B7+antiCD3 engineered particles were produced from genetic-modified host cell lines. Host cells were surface modified by genetic expression of CD80/B7.1, CD86/B7.2, and/or antiCD3 single-chain antibody (antiCD3sFv) by retroviral transduction. Human CD80/B7.1 gene cDNA was cloned from peripheral blood mononuclear cell (PBMC) RNA amplified by reverse-transcriptase polymerase chain reaction (RT-PCR) using synthetic oligonucleotides (5'-primer: 5'-GATC TCTAGACTGCC ATGGGCCACACACGG-3' and 3'-primer: 5'-GATC GTCGACCTTCTGCGGACACTG TTATACAG-3'). This PCR fragment overlaps protein initiation and termination sites with Xba1 and Sal1 restriction enzyme sites inserted at each end, respectively for cloning purposes. This 867 nucleotide fragment was cloned into an encephalomyocarditis virus internal ribosomal entry site (IRES) motif [28] and then into pN2*neo vector [29] a MuLV neomycin phosphotransferase (neo)-containing murine retroviral vector, resulting in pJDMT6 plasmid construction (Fig. 1). Human CD86/B7.2 cDNA was cloned from PBMC RNA amplified by RT-PCR using synthetic oligonucleotides O-JDMT61 (5'-primer: 5'-GATC CTCGAGGTCACAGCAGAAGCAGCCAAA ATGG-3') and O-JDMT62 (3'-primer: 5'-GATC GTCGACGGGCTTTACTCTTTAA TTAAAAACATG-3'). The resulting PCR fragment overlaps the protein initiation and termination sites with Xho1 and Sal1 restriction enzyme sites inserted at each end, respectively for cloning purposes. The 1026 nucleotide fragment was then cloned into pCGII plasmid (Invitrogen, San Diego, CA) and then into pJM573neo [30] (a MuLV retroviral vector containing an IRES-neo cassette) replacing the enhanced green fluorescent protein (eGFP) gene, resulting in the pJDMT19 plasmid construction (Fig. 1). The antiCD3 single-chain gene portion within the pα CD3env plasmid (a kind gift from Dr. Stephen J. Russell at the Mayo Foundation) was amplified using RT-PCR using synthetic oligonucleotides O-JDMT4204 (5'-primer: 5'-GCAT GGGCCCCGGCC ATGGCCCAGGTG-3') and O-JDMT4205 (3'-primer: 5'-GCAT GTCGACTGCGGCCGCCCG TTTGAT-3'). This PCR fragment overlaps the protein initiation and termination sites with Apa1 and Sal1 restriction enzyme sites inserted at each end, respectively for cloning purposes. The 751 nucleotide fragment was cloned into a modified pHook-3 vector (Invitrogen, San Diego, CA), then the single-chain surface-expressed antibody cassette was removed and placed into an Apa1 minus pBluescript SK plasmid (Stratagene, San Diego, CA). The phOxsFv antibody sequence was replaced with the antiCD3-sFv sequence and the entire murine-Ig signal peptide-antiCD3sFv-PDGF transmembrane domain cassette was placed into pJDMT45 (a MuLV retroviral vector containing an IRES-zeocin cassette), resulting in pJDMT-50 plasmid construction (Fig. 1). The retroviral vector, pJDMT45 is identical to pJM573neo except that the neo gene is replaced with the zeocin gene as a drug selectable marker for selection of vector transduced cells. All PCR amplified fragments were DNA sequenced before cloning into their respective plasmids.

The retroviral vectors pJDMT6, pJDMT19, and pJDMT50 were transfected into GP+E-86 ecotropic producer cells [31] [ATCC No. CRL-9642] and amphotropic retrovirus was prepared by transducing PA317 cells [32] [ATCC No. CRL-9078] twice with the ecotropic virus as described [30]. Titers of pJDMT6, pJDMT19, and pJDMT50 retroviruses are 1.2 × 10^6^, 6.4 × 10^5^, and 1.0 × 10^6 ^colony-forming units/ml, respectively. All retrovirus supernatants were free of helper virus. Stable retroviral transduced cells were enriched by drug selection using G418 (1.0 mg/ml) for neomycin-containing vectors and zeocin (0.2 mg/ml) for zeocin-containing vectors. Centrifugal procedures (1,650 g for 1 h) were used to viral transduce Lof(11-10), MDCK, and 1119 cells; these procedures were adapted from experiments done with human mesenchymal adult stem cells as described [29]. Transduction efficiency was accessed by drug-resistant colony formation. Two successive cycles of transduction further enhanced gene expression and was done routinely.

### Particle formulation: infection, expansion, harvest, concentration, and inactivation

Intact infectious viral particles are produced by either acute infection of cell lines (for HSV and Influenza) or from chronic-expressing cell lines (for HIV). Lof(11-10) cells were infected with HSV-2 strain G; monolayer cultures were exposed to HSV-2 for 1 hour in minimal volume to cover the cell layer. MDCK cells were infected with Influenza strains (Japan), (Taiwan), and (PR8). All serum was removed from the cell monolayers; the virus was added for 1 hour at 37°C with gentle motion; and replaced with serum-free DMEM media containing 0.01% trypsin. Although expansion of HSV- and Influenza-based particles are limited to the number of initial cultures established, expansion of HIV-based particles is easily preformed infinitely by the addition of fresh culture media. HIV-based particle cultures are routinely expanded 1:3. HIV particle released from expanded cultures were further enhanced 100-fold by TPA (1.0 pg/ml) and TNF-alpha (25 ug/ml) treatment [33,34] 2 days before culture media collection. For HSV and Influenza cultures, supernatants are harvested between 1 and 3 days based on the time needed for cellular cytopathic effect to approach 100%. Influenza titers are monitored by agglutination assays using chicken red blood cells for titer determination. In all cases, culture supernatants were clarified by two-centrifugations; the first at 1,200 rpms and the second at 4,000 rpms in a tabletop refrigerated centrifuge. Although some experiments used particle preparations collected directly from clarified culture supernatants, other particle preparations were concentrated by polyethylene glycol (PEG)-precipitation. Concentration (25- to 200-fold) is performed by the addition of 1.2 g of PEG (MW = 3,350) and 2.2 g of NaCl per 100 ml of culture supernatants, resulting in a 6% final concentration that would favor the recovery of particles relative to free proteins. Once dissolved, the preparation is stored at 4°C overnight and the precipitate is collected by centrifugation at 4,000 rpm for 45 minutes in a refrigerated tabletop centrifuge. Centrifuge tubes were inverted to remove as much of the supernatant as possible and the precipitated material was resuspended in buffer containing 0.002% Tween-80. In some experiments, conditioned media were tested as lyophilized preparations; 5 ml media aliquots were transferred to 15 ml conical tubes and placed under a vacuum in a desiccator until completely dried. Lyophilized preparations were stored at room temperature; non-lyophilized preparations (conditioned media and PEG-concentrated) were stored at -80°C. Inactivation of viral infectivity was done by the addition of 1 mM aminomethyltrimethyl psoralen (AMT) followed by UV-irradiation (3 J/cm^2^); the AMT-UV treatment cross-links nucleic acid containing molecules thereby inactivating viral replication without affecting intact protein structure [35].

### Particle addition and infectious HIV challenge

Primary PBL cultures were initiated at 5 to 10 × 10^6 ^cells/ml in RMPI supplemented with 10% heat-inactivated fetal calf serum. The primary cells were exposed to engineered and non-engineered particles (1 to 15 ug/ml for HIV, HSV, and Influenza; 50 to 600 ng/ml of p24 for HIV) at the initiation of the culture, day 0. For experiments using culture supernatant containing particles (conditioned media), 1 to 2 milliliter were added to every 5 ml of culture media; for concentrated (25- to 200-fold) particles (PEG-concentrated), 1 to 5 microliter were added to each milliliter of culture media. Primary cell cultures were setup in T-25 flasks and aliquots removed for assay at the indicated time points. Only in HIV infectious viral challenge experiments is phytohemaglutinin (PHA) and IL-2 (PHA/IL-2) added at the same time as the particles. No particle (control) cultures receive PHA/IL-2 alone; the concentration of PHA was 10 ug/ml and IL-2 was 100 units/ml. In the case of HIV infection, primary cells are exposed to media containing PHA/IL-2 to ensure maximal conditions for infectious HIV replication. After 3 days, cultures were centrifuged and resuspended in appropriately diluted infectious HIV preparations for 2 hours. The cultures were rinsed with phosphate-buffered saline 3 times to ensure the removal of all unbound p24 antigen that was introduced by the addition of HIV-based particles and the infectious HIV preparation. The cultures were maintained in media supplemented with IL-2.

### Preparation of PBMCs and PBLs

Sixteen different Donor cells are used in the experiments outlined in this report. Healthy human donor leukopheresis preparations were purchased from the American Red Cross (Rockville, MD). PBMCs/PBLs were prepared by density gradient centrifugation over Ficoll-Hypaque according to the manufacturer's instructions (Amersham-Pharmacia Biotech, Piscataway, NJ). All experimental results were obtained on freshly isolated PBMCs that were monocyte depleted by countercurrent centrifugal elutriation (enriched lymphocyte preparation-PBLs). The monocyte depleted PBL cell populations are enriched for CD4, CD8 T cell and B cell lymphocytes (data not shown).

### Proliferation assay

A one-step non-radioactive assay using Alamar Blue (Invitrogen, Carlsbad, CA) was used to monitor T cell activation [36]. Cell aliquots (1 to 2 × 10^5 ^cells) were removed from T-25 culture flasks and placed in 96-well panels containing 10 ul of Alamar Blue. Incubation at 37°C was continued and plates were read within 24 hours on a Cytofluor 4000 fluorescence plate reader (Applied Biosystems, Foster City, CA). All values are the average of samples done in triplicate and the relative values are averaged with standard deviation between 2 to 5%.

### Antibody detection

Panels of 96-well plates were coated with detergent disrupted virions (lysates) obtained from purified virus preparations (Advanced Biotechnologies Inc., Columbia, MD). In some cases, cellular lysates were used. PBL cultures were treated as indicated and at the various time points an aliquot of cells (1 to 2 × 10^6 ^cells) was removed and placed in the lysate coated 96-well plates in triplicate. After incubation for 3 days, the 96-well plates were washed and incubated with anti-human conjugated horseradish peroxidase antibody. Detection of bound human antibodies was performed using an Bio-Rad, Model 3550 Microplate Reader (Richmond, CA).

### Cytokine detection: IFN-γ; IL-10; IL-4

The measurement of cytokines was done by ELISA. Supernatants from cultures were collected at intervals after particle stimulation and assayed in duplicate for the presence of IFN-γ, IL-10, and IL-4 using Predicta™ cytokine kits (Genzyme Diagnostics, Cambridge, MA). The manufacturer's protocol was followed for each kit. Optical density at 450 nm was read on a Bio-Rad, Model 3550 Microplate Reader (Richmond, CA) and the cytokine concentration is determined from the standard curve.

### Fluorescence-activated cell sorting analysis

Analysis of cell surface molecules was performed using a panel of fluorochrome-labeled monoclonal antibodies diluted according to the manufacturer's instructions (BD Biosciences Pharmingen, San Diego, CA). Nonspecific fluorescence was determined by substitution with appropriate isotype-matched irrelevant monoclonal antibodies. Data were analyzed by collecting 10,000 events on a Becton Dickinson Vantage instrument using Cell-Quest software.

## Competing interests

JDM has personal and financial relationships with the company's organization in the form of stock and patent-pending inventorship related to the technology.
